# Intentional Discontinuation of Psychostimulants Used to Treat ADHD in Youth: A Review and Analysis

**DOI:** 10.3389/fpsyt.2021.642798

**Published:** 2021-04-20

**Authors:** W. David Lohr, Jonathon W. Wanta, Megan Baker, Eugene Grudnikoff, Wynne Morgan, Divya Chhabra, Terry Lee

**Affiliations:** ^1^Division of Child and Adolescent Psychiatry, Department of Pediatrics, University of Louisville, Louisville, KY, United States; ^2^Department of Psychiatry and Behavioral Sciences, University of Washington, Seattle, WA, United States; ^3^Momentum for Mental Health, Palo Alto, CA, United States; ^4^School of Medicine, Hofstra University, Hempstead, NY, United States; ^5^Division of Child and Adolescent Psychiatry, University of Massachusetts Medical School, Worcester, MA, United States; ^6^Department of Psychiatry, New York-Presbyterian Hospital, Columbia University College of Physicians and Surgeons, Weill Cornell Medical College, New York, NY, United States

**Keywords:** discontinuation, psychostimulants, ADHD, youth, intentional, evidence

## Abstract

**Objectives:** This paper reviews the literature on intentional discontinuation of psychostimulants in ADHD to summarize what is known about clinical course of controlled discontinuation and guide practitioners who are considering stopping these medications for youth with ADHD.

**Methods:** A systematic search was executed in Cochrane CENTRAL, EMBASE, Psychinfo, and MEDLINE databases to identify all articles that addressed the topic of deprescribing of psychotropic medications in children and adolescents. Keywords and search strings were developed using “PICO” framework, involving Population of interest (<18 y.o.), Intervention (“discontinuation,” “deprescribing,” and synonyms), Comparator (continuation of specific medications), and Outcomes. Ten reviewers conducted the initial screen *via* a single reviewer system. Articles that met a set of three inclusionary criteria were selected for full text review and identification as specific to discontinuation of stimulants in ADHD.

**Results:** The literature review identified 35 articles specifically addressing intentional deprescribing, discontinuation, tapering, or withdrawal of stimulants for children and adolescents with ADHD. In addition to providing broad support for the efficacy of stimulants to treat ADHD and reduce negative outcomes, there is a distinct population of children and adolescents with ADHD who do not relapse or deteriorate when taken off medications for ADHD. The majority of articles addressed either the re-emergence of ADHD symptoms or side effects, both desired and adverse, following discontinuation of stimulants. While confirming the ability of stimulants to treat ADHD in youth, our results support periodic consideration of trials of stopping medications to determine continued need.

**Conclusions:** This systematic review summarizes the literature on deprescribing stimulants for ADHD in children and adolescents. Further research is needed to determine the optimal duration of treatment, identify patients that may benefit from medication discontinuation, and inform evidence-based guidelines for discontinuation when appropriate. More research is needed to understand and define the subgroup of youth who may succeed with stimulant discontinuation.

## Introduction/Objectives

Attention-deficit/hyperactivity disorder (ADHD) is a common childhood psychiatric condition. It is generally considered a long-term condition with up to two-thirds of individuals diagnosed in childhood continuing to experience the condition or symptoms in adulthood (1–3). Research on effective pharmacotherapy of ADHD is largely short-term spanning weeks, with some large randomized controlled trials (RCTs) lasting beyond a year, and prospective data sometimes several years (4). This contrasts with clinical practice, where patients can be treated for longer durations, sometimes decades or beyond.

Psychostimulants are the medications with the highest established efficacy in treating youth with ADHD and are recommended as first-line treatment options (5–7). While the efficacy of stimulants is well-established, optimal duration of treatment and effects of medication discontinuation are less well-characterized (4). Many individuals diagnosed with ADHD, their families, providers, and other stakeholders understandably have questions about how long they should continue on medications as they consider the risks and benefits of medication discontinuation vs. continuation.

These questions have added pertinence as rates of psychiatric medication prescriptions have increased dramatically in the child and adolescent population over the last 20 years (8). In addition, the growth of psychotropic polypharmacy in children and adolescents has raised concerns given the lack of evidence to document efficacy and safety (9). This awareness has given rise to a new medical literature on deprescribing and/or discontinuing of psychotropic medications.

Deprescribing is a structured approach to identifying and discontinuing medications when existing or potential harms outweigh existing or potential benefits. Such deprescribing may be motivated by a variety of reasons, not limited to concerns about polypharmacy, managing adverse effects, changing evidence base or best practices, changing clinical need, or patient preference (10). First introduced in the geriatric population, deprescribing has since been applied to the fields of general and eventually child and adolescent psychiatry (10–12). This process is complicated by generally inadequate evidence to inform the optimal duration of pharmacotherapy, the risks and benefits of medication discontinuation vs. continuation, and standardized processes for tapering and eventual medication discontinuation.

In clinical practice, medication may be discontinued by either patient or provider for many reasons, at times due to adverse effects, lack of symptom control, client or family preference, when diagnostic formulation changes, changing health status, or when it is believed medication may no longer be necessary to maintain functioning (13). This paper reviews the literature on intentional discontinuation by providers of psychostimulants in ADHD to summarize what is known about clinical course of controlled stimulant discontinuation and guide practitioners who are considering, within the process of deprescribing, stopping psychostimulants for youth with ADHD.

## Methods

This effort was initiated by the American Academy of Child and Adolescent Psychiatry (AACAP) Adoption and Foster Care Committee to develop a resource on deprescribing psychotropic medications in youth. A systematic search was executed in Cochrane CENTRAL, EMBASE, Psychinfo, and MEDLINE databases to identify all articles that addressed the topic of deprescribing of psychotropic medications in children and adolescents. Keywords and search strings were developed using “PICO” framework, involving Population of interest (<18-year-olds), Intervention (“discontinuation,” “deprescribing,” and synonyms), Comparator (continuation of specific medications), and Outcomes. Ten reviewers conducted the initial records screen of title and abstract via a single reviewer system.

Given the large number of articles to screen, exclusion by a single reviewer was allowed at this step. Reviewers could choose to include, exclude, or tag the article for a second review. In the latter case, two reviewers (WM and MB) reviewed the title, journal, and abstract. Both reviewers were required to agree on either inclusion or exclusion, with discordant responses addressed in conversation. Articles included at this step were identified for full-text review. Full-text review included review of references to detect any potentially relevant articles that had not be identified in the initial database search, and pertinent citations were identified and added to the initial screening step for full review. Additionally, articles were similarly added if identified by reviewers from other mechanisms such as reading of literature or suggestions from experts in the field.

There were three inclusionary criteria, and included studies met all three criteria. First, the topic of the article relates to provider initiated deprescribing, discontinuation, tapering, withdrawal, or reduction of psychiatric medications. Studies focused only on patient non-adherence were not included. Additionally, the article included a psychotropic medication with behavioral health/mental health indications (not inclusive of supplements and minerals). Medications with psychiatric indications (e.g., clonazepam) being studied for non-psychiatric reasons (e.g., seizures) were included if the outcome measure was pertinent to psychiatry (cognition), as opposed to only neurological (seizure relapse). Finally, the population studied was <18 years of age, or if spanning youth and adult populations, the <18-year-old subset is analyzed independently.

**Figure 1:**
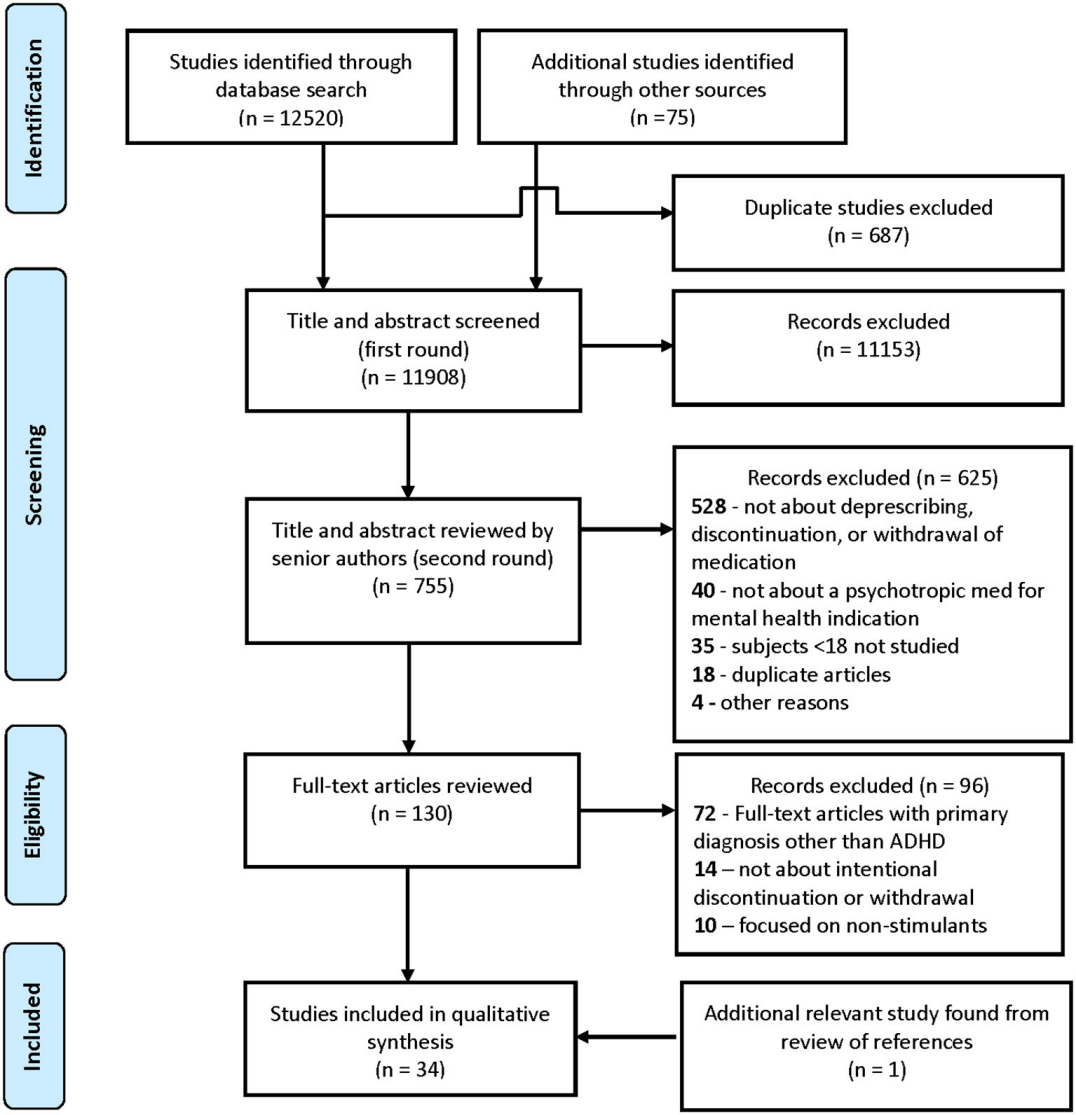
Process identification of studies, screening, and eligibility flow diagram for discontinuation of stimulants in children with ADHD.

The initial search returned 12,520 citations. An additional 75 (46 new articles and 29 duplicates) references were identified via review of reference or authors knowledge of pertinent studies, including those published after the initial search that met inclusion criteria and came to authors' awareness (see Figure 1).

We identified 58 articles specific to ADHD as a primary diagnosis had a second full-text review by two authors (WL and JW) to identify themes and data pertinent to intentional discontinuation, tapering, or withdrawal of medications treating ADHD. Studies relating to medication non-adherence or discontinuation without physician involvement were excluded (*N* = 14), as were those focusing on non-stimulants (*N* = 10). Discontinuation case studies were excluded if the primary diagnosis was not ADHD. Case studies that described side effects that emerged in the process of discontinuation were included. Additionally, a hand search of the bibliographies of full text articles and updates from the published literature yielded one additional study.

## Results

The literature review identified 35 articles specifically addressing intentional deprescribing, discontinuation, tapering, or withdrawal of stimulants for children and adolescents with ADHD. Our review covered 15 case reports, 3 clinical guidelines, 2 literature reviews, 2 observational studies, and 13 RCTs.

We identified 13 RCTs that systematically investigated the effects of discontinuing stimulants in children and adolescents with ADHD (Table 1). Of these, seven addressed the re-emergence of ADHD symptoms following discontinuation of stimulant monotherapy, four for methylphenidate derivatives and three for amphetamine derivatives. Three RCTs investigated the discontinuation of stimulants administered with concomitant cognitive or attention control therapies. Just one RCT studied side effects, namely, re-emergence of tics, with discontinuation of a stimulant. One RCT each addressed the effects of weekend or summer stimulant holidays on ADHD symptoms and medication side effects.

**Table 1:** Randomized controlled trials that address discontinuation of psychostimulants in children with ADHD.

**Reference**	**Industry sponsored**	**Duration of medication discontinuation**	**Total *N***	**Age (years)**	**Number of boys (% of total)**	**Medication(s)**	**Primary outcome measure(s)**	**Findings**
Abikoff et al. (14)	No	4 weeks	50	6–12	45 (90%)	MPH, dextroamphetamine, pemoline	CTRS BRS Home Hyperactivity Scale Parent Attitude Test Standardized Tests of achievement and cognition	The combination of a stimulant with cognitive training did not facilitate stimulant withdrawal
Abikoff et al. (15)	Yes	1 year	103	7–9.9	(93%)	MPH	CPRS Home Situations Questionnaire CTRS School Situations Questionnaire	All children relapsed when switched to placebo (single-blind), mean 8.6 vs. 17.1 days for MPH alone vs. MPH with multimodal psychosocial treatments, none of the parent, teacher, or psychiatrist evaluations yielded significant group or interaction effects.
Arnold et al. (16)	Yes	2 weeks	75	6–16	61 (81%)	Dexmethylphenidate	CGI-I SNAP-ADHD	Placebo group 3.6 × more likely to “fail” treatment (CGI-I 6 or 7); statistically significant deterioration on Teacher and Parent SNAP scales compared to continued medication.
Banaschewski et al. (17)^*^	Yes	6 weeks	153	6–17	119 (78%)	Lisdexamfetamine	CHIP-CE: PRF WFIRS-P	The improvement in health-related quality of life and functional status during the lead-in phase was maintained in the lisdexamfetamine arm while those in the placebo arm had statistically significant deterioration for both.
Brown et al. (18)	No	24 h to 1 week	35	5–14	28 (80%)	MPH	ACRS CPRS Various tests of attentional deployment and cognitive style, academic achievement	Neither the combination of a stimulant with cognitive therapy nor a stimulant with attention control therapy for 3 months facilitated stimulant withdrawal
Coghill et al. (17)^*^	Yes	6 weeks	153	6–17	119 (78%)	Lisdexamfetamine	ADHD-RS	Rates of treatment failure were 15.8% in the lisdexamfetamine group and 67.5% in the placebo group. Median time to treatment failure was 17 days for the placebo group.
Gillberg et al. (19)	No	12 months double-blind treatment followed by 3 months of single-blind placebo in both groups. Placebo was tapered over 2 weeks.	62	6–11	51 (82%)	Amphetamine	CTRS	The improvement in Conners Teacher and Parent Rating Scale scores during the lead-in phase was maintained in the amphetamine arm while those in the placebo arm had significant deterioration for both; 71% of those in the placebo group withdrew or went into open treatment compared to 29% of those in the amphetamine group.
Klein et al. (20)	No	Stimulant holiday for 3 months over the summer for two consecutive summers	58	6–12	53 (91%)	MPH	Height Weight	At the end of the first summer, the group that had been discontinued from stimulants weighed on average 0.9 kg more than the treatment group; there was no statistically significant difference in height. At the end of the second summer, the group that had been discontinued from stimulants was on average 1.5 cm taller than the treatment group; there was no longer a statistically significant difference in weight.
Martins et al. (21)	No (medication supplied by industry)	Stimulant holiday for four weekends	40	Mean age 9.0 and 9.6 for MPH and placebo group, respectively	40 (100%)	MPH	ACRS SERS	There was no difference between groups, suggesting a lack of rebound ADHD symptoms during a short-term MPH weekend discontinuation. The weekend placebo group had significant reduction in insomnia reported, and there was a trend toward reduction in decreased appetite.
Matthijssen et al. (22)	No	3-week taper, 4-week discontinuation	94	8–18	73 (78%)	MPH	ADHD-RS CGI-I	Mean scores favored the group that continued MPH treatment on the ADHD-RS. 40% of those who discontinued medication worsened on the CGI-I compared to 16% of those who continued medication.
Nolan et al. (23)	No	2-week crossover	19	6–17	18 (95%)	MPH, dextroamphetamine	Various measures of tics and ADHD symptoms	No significant withdrawal effect on tics with placebo. Significant increase in some parent-reported behavioral symptoms, hyperactivity, and aggression while on placebo.
Waxmonsky et al. (24)	No	Weekend drug holidays	71	5–12		MPH	Weight, height, CGI-S, growth trajectories	Medication use was associated with reductions in height and weight, caloric supplement and drug holidays increase weight velocity more than monitoring
Zeiner et al. (25)	No	3 weeks	21	7–12	21 (100%)	MPH	**PACS** CTRS Neuropsychological testing	76% of boys had a significant worsening in behavioral problems either at home or at school while on placebo

* *One study with two resultant papers*.

*ACRS, Abbreviated Conners Rating Scale; ADHD-RS, ADHD Rating Scale-IV; BRS, Hillside Behavior Rating Scale; CGI, Clinical Global Impression (Severity/Improvement); CHIP-CE: PRF, Child Health and Illness Profile—Child Edition: Parent Report Form; CTRS, Conners Teacher Rating Scale; MPH, methylphenidate; PACS, Parental Account of Childhood Symptoms; WFIRS-P, Weiss Functional Impairment Rating Scale-Parent Report*.

### Re-emergence of ADHD Symptoms Following Stimulant Discontinuation

The seven studies below were designed to address the efficacy of short- and long-term use of stimulants for ADHD. However, the studies were included in this review as they all include a randomized placebo-controlled double blind discontinuation arm. While most children experience rapid re-emergence of ADHD symptoms following stimulant discontinuation, there is a subset of the population, ~30%, who do not relapse or deteriorate when taken off their stimulant. We now look at each study in detail.

Following a 3-month single-blind amphetamine titration period, Gillberg et al. randomized 62 children aged 6–11 to continued treatment or taper to placebo for 12 months in a double-blinded manner, followed by 3 months of single-blind placebo for those continued on amphetamine (19). During the randomized withdrawal phase, all-cause discontinuation was 71% in the placebo group compared to 29% of the amphetamine group, most often within the first 3 months of randomization. The improvement in Conners Teacher and Parent Rating Scale scores during the lead-in phase was maintained in the amphetamine arm while those in the placebo arm had significant deterioration, without difference between older (9–11) than younger (6–8) cohorts. Youth randomized to continued amphetamine were withdrawn in a single-blind fashion at month 15 without significant change in parent or teacher scores at 18 months. Sex, comorbid diagnoses, and WISC scores at baseline did not contribute to outcomes. The study is notable for the long duration of double-blind exposure to stimulant or placebo.

Nolan et al. systemically studied stimulant discontinuation in 19 youth aged 6–17 years with comorbid ADHD and a tic disorder, the majority (*N* = 17) on MPH and two on dextroamphetamine (23). Children were continued on their home medication for 2 weeks, then randomized to placebo or continued treatment for 2 weeks, and then subsequently to the alternate condition, in a placebo-controlled crossover design. Primary outcomes were measures on the quantity and quality of tics (discussed further below). Secondary outcome measures related to ADHD noted significant increase in some parent-reported behavioral symptoms, hyperactivity, and aggression, suggesting continued efficacy of stimulants, though rates of relapse were not reported, and the clinical significance of the findings are not stated. Children had poorer performance on classroom-simulated tests of attention but not dyscontrol or impulsivity.

A study by Zeiner recruited 21 boys aged 9–13 years with a mean MPH prescription duration of 1.75 years (25). The boys were randomized in a double-blind, placebo-controlled, crossover design to MPH or placebo for 3 weeks with a 1-week wash-out period between study arms. As a group, there was a statistically significant increase in both hyperactive and defiant behavior problems at school during the placebo arm and an increase in defiant (but not hyperactive) behavior at home. On the individual level, 76% of boys had a significant worsening in behavioral problems either at home or at school. Based on home ratings, about 40% of boys were the same or better on placebo than methylphenidate, though for school rating, this was only 10%. There was no correlation with age, IQ, or behavioral problems to predict who might fare better or worse during the placebo arm. Attention measures were affected more than measures of impulsivity. This study highlights the importance of feedback from multiple settings when determining impact of medication discontinuation. Authors suggested a protocol for trial discontinuation, starting with a brief 1-week discontinuation, and if results are ambiguous, then placebo substitution for 3–4 weeks.

A large RCT evaluating the withdrawal of dexmethylphenidate utilized a 6-week open-label dose titration lead-in, with 75 “responders” (CGI-I of 1 or 2) proceeding to a 2-week randomized, double-blind, placebo-controlled withdrawal phase (16). The improvement in CGI-I and Teacher and Parent SNAP-ADHD scores during the lead-in phase was maintained in the dexmethylphenidate arm while those in the placebo arm had significant deterioration for both. The proportion of “treatment failures” (≥6 on the CGI-I) was 61.5% in the placebo group and 17.1% in the dexmethylphenidate group. Redefining treatment failure (CGI-I score of ≥5) led to rates of 71.8% of the placebo group and 45.8% of ongoing medication. Those in the placebo arm also had significantly worse ratings on teacher-rated SNAP, parent-rated SNAP (3 pm and 6 pm), and math testing. No treatment-related serious adverse events or withdrawal symptoms were noted. Given that inclusion required initial response to stimulants during the open-label phase, it is surprising that nearly immediate discontinuation did not result in worsening in over 1/3 of youth, which may represent placebo responders; authors did not comment on specific patient demographics that contributed to treatment failure.

One study with two resultant papers studied the effects of lisdexamfetamine discontinuation in a large, majority European, multi-center study. Youth aged 6–17 enrolled first into a 26-week open-label lead-in with lisdexamfetamine; completers (*N* = 157) then enrolled in a 6-week randomized double-blind placebo-controlled withdrawal study. The primary outcome in Coghill et al. was treatment failure, defined as >50% increase in ADHD-RS total score and ≥2-point increase in the CGI-S score at any single assessment point during the withdrawal period. Rates of treatment failure were 15.8% in the lisdexamfetamine group and 67.5% in the placebo group (17). Most of those who met criteria for treatment failure did so within the first 2 weeks; the median time to treatment failure was 17 days for the placebo group. One study limitation is that medication was withdrawn abruptly, and treatment failure could be met at a single time point, so rebound or withdrawal effects could have led to early treatment failure. No serious treatment emergent adverse events were reported during the randomized withdrawal phase. Subsequent analysis showed improvement in health-related quality of life and functional status during the lead-in phase and then maintained in the lisdexamfetamine arm while those in the placebo arm had significant deterioration for both (26).

In the most recent and best-designed study, Matthijssen et al. conducted a randomized, placebo-controlled discontinuation study to characterize the ongoing benefit of methylphenidate and withdrawal effects of discontinuation (22). They enrolled 94 youth aged 8–18 who had received MPH consistently for more than >2 years. For those assigned to placebo, medication was tapered in a stepwise fashion over 3 weeks and then discontinued for 4 weeks. After 7 weeks, mean scores favored the group that continued MPH treatment; effects size (*d* = −0.23) and absolute difference in total scale score (<3 points) were small. Differences were significant for ADHD-RS total score and inattention subscale score, but not hyperactivity–impulsivity subscale score. Differences were significant only for the younger cohort (below median age of 13.8), and when applied to an older subset of youth, no difference was noted. For the secondary outcome measure CGI-I, those who discontinued medication worsened about 40% of the time, compared to nearly 16% in those who continued medication. More participants dropped out due to worsened functioning in the placebo group, though all were included in the analysis above. No serious adverse events were noted; change in appetite and weight change were more commonly reported with medication discontinuation. Authors concluded that a subset of youth was discontinued from methylphenidate without exacerbation of ADHD symptoms and that their data support existing guidelines recommending trial discontinuation on a periodic basis. One limitation is that authors noted that many qualifying participants who declined to participate cited ongoing benefit from MPH, suggesting that the study population may have been biased toward those suspecting limited benefit from ongoing medication and wanting trial discontinuation.

### Re-emergence of ADHD Symptoms Following Stimulant Discontinuation After Non-pharmacologic Interventions

Three RCTs described were designed to determine if concomitant stimulant use with various therapy regimens attenuated ADHD symptom re-emergence when the stimulant was discontinued. Researchers found no difference in youth who received up to 1 year of non-pharmacologic interventions when stimulants were discontinued. Each trial is described in more detail below.

The first study randomized 50 children to continue their home stimulants as usual (MPH, dextroamphetamine, or pemoline), home stimulants with cognitive training, or home stimulants with attention control for 16 weeks before switching to placebo (14). The authors conclude that the combination of a stimulant with cognitive training did not facilitate stimulant withdrawal, as there was no statistically significant difference in the number of subjects who required restarting a stimulant during the placebo phase (77 to 90%), nor was there a difference in number of days subjects were able to tolerate placebo before necessitating stimulant re-prescribing (mean 14.9 to 18.2 days). There were few measures that favored children who had been in the stimulant-only arm. For most measures, there were no differences in the three treatment arms when children were discontinued from their stimulants.

A similar study randomized 34 children to 3 months of cognitive training plus MPH, attention training plus MPH, cognitive training plus placebo, or attention training plus placebo (18). In the two treatment arms in which children received MPH, the stimulant was discontinued 24 to 72 h prior to post-testing. Researchers found no difference in multiple tests of attention, academic achievement, behavior at home, or ADHD and conduct symptoms at school, leading authors to conclude that children must remain on medication to sustain improvements observed with methylphenidate.

The largest and most robust RCT addressing the effect of non-pharmacologic intervention on discontinuation of a stimulant randomized a group of 103 children to 1 year of MPH, MPH plus multimodal psychosocial treatment (including parent training and counseling, social skills training, psychotherapy, and academic assistance), or MPH plus attention control psychosocial treatment (15). After a year, subjects were discontinued on their stimulants and started on placebo in a single-blind manner. All children relapsed when switched to placebo and quickly required re-prescribing of MPH. Mean duration of the placebo trials was 8.6 days for MPH alone, 17.1 days for MPH with multimodal psychosocial treatments, and 11.7 days for MPH with attention control psychosocial treatment, which is statistically significant but of uncertain clinical significance. None of the parent, teacher, or psychiatrist evaluations yielded significant group or interaction effects.

### Mitigation of Adverse Effects With Discontinuation of Stimulants

Some auxiliary data can be gleaned from the RCTs on stimulant discontinuation in children with ADHD. A common motivation for medication discontinuation is adverse effects. Stimulants are well-known to contribute to weight loss and blood pressure; it is also hypothesized that stimulant withdrawal can exacerbate tics in at-risk youth. The RCTs with a more adverse effect focus show nominal but often statistically benefit when stimulants are discontinued.

Within these RCTs, there was evidence for and against mitigation of adverse effects when stimulants are discontinued. Weight loss, one of the most common side effects of stimulants, was measured throughout the Coghill et al. trial (17). During the 6-week randomized double-blind placebo-controlled discontinuation of lisdexamfetamine, patients who continued to receive the treatment maintained a stable weight, whereas those who were randomized to placebo increased in weight.

A second study noted that abrupt discontinuation of dexmethylphenidate was not associated with rebound or withdrawal symptoms (16). In those that continued medication, blood pressure rise was nominally lower (SBP 2.6 ± 11.6 vs. 3.1 ± 9.5) and increase in heart rate was higher (5.4 ± 12.2 vs. 1.9 ± 10.7) than in the placebo group.

Finally, one study examined if stimulant discontinuation would exacerbate or improve tics in those with premorbid chronic motor tic disorder or Tourette's disorder (23). Based on parent report, clinician report, and direct observation in a simulated classroom, there was “little evidence” that stimulant discontinuation caused tic exacerbation. The only tic-based measure that reached clinical significance was the clinician's 2-min Vocal Tic count, which demonstrated a mean of 1.2 tics in the blinded treatment group and 0.4 tics in the blinded placebo group (*p* = 0.0037).

### How Do Summer or Weekend “Stimulant Holidays” Relate to Long-Term Stimulant Discontinuation?

Three additional RCTs were included in this review that addresses summer and weekend stimulant holidays. While the clinical goals of “stimulant holidays” may not ostensibly coincide with stimulant discontinuation, there is nonetheless clinically relevant data to be gleaned. The below studies support the use of “stimulant holidays” to manage common stimulant side effects without clinical deterioration.

Fifty-eight children on long-term MPH for ADHD were randomly assigned to continue their stimulants throughout the summer or to discontinue for two consecutive summers (20). At baseline, there was no difference in the two groups for age, height, or weight. At the end of the first summer, the group that had been discontinued from stimulants weighed on average 0.9 kg more than the treatment group; there was no statistically significant difference in height. At the end of the second summer, the group that had been discontinued from stimulants was on average 1.5 cm taller than the treatment group; there was no longer a statistically significant difference in weight. This was one of the first studies to link periodic discontinuation of stimulants with benefit to height that have been replicated over the years.

A more restrictive “stimulant holiday” can be found in a double-blind placebo-controlled RCT (21). The 40 boys who entered the study were titrated on MPH from 0.3 mg/kg/day to 0.7 mg/kg/day as tolerated to target ADHD symptoms. Subjects were then randomized to continued MPH or placebo on the weekends to mimic weekend “stimulant holidays.” Conners' Abbreviated Rating Scales were administered to teachers and parents on Mondays. Teachers were instructed to assess behaviors for the given Monday, and parents were instructed to assess behaviors for the given weekend. There was no difference between groups, suggesting a lack of rebound ADHD symptoms during a short-term MPH weekend discontinuation. It is also possible that the onset of MPH was rapid enough such that teachers could not appreciate a difference in behaviors with or without the weekend holiday. Importantly, the weekend placebo group had significant reduction in insomnia reported, and there was a trend toward reduction in decreased appetite.

Finally, a recent RCT examined weekend drug holidays as a weight recovery treatment and found this practice along with caloric supplementation and increased monitoring effective in increasing weight velocity in children taking stimulants (24). Adherence to drug holidays was high over the 30-month study duration (95%); in fact, many parents did not give weekend medication even when asked to. Effects on height were not seen.

### Observational Studies

Two observational studies assessed effects of stopping stimulants, the first on neuropsychological testing performance and the second on ADHD symptoms (Table 2). Both had significant methodological limitations.

**Table 2:** Observational studies that address discontinuation of psychostimulants in children with ADHD.

**Reference**	**Industry sponsored**	**Duration of medication discontinuation**	**Total *N***	**Age**	**Number of boys (% of total)**	**Medication(s)**	**Primary outcome measure(s)**	**Findings**
Hoare and Sevar (27)	NR	>24 h	15	4.5–14.6	100%	MPH	Primary: CANTAB SNAP-IV	Compared to testing with MPH, youth performed worse on placebo for some test measures (pattern recognition, spatial working memory, intra/extra dimensional set-shifting) primarily relating to executive function, though no effect was noted on other measures. All except one youth had worse SNAP scores on placebo.
Sleator (28)	NR	“Within patient” design, 1 month each of three different drug doses and PBO	42; only 28 received PBO trial	NR	NR	MPH	Conners' teacher's abbreviated symptom questionnaire (ASQ) remission defined as <15	17/42 (40%) showed significant deterioration during PBO; 11/41 (26%) showed no deterioration on PBO

*CANTAB, Cambridge Neuropsychological Test Automated Battery; Conners' ASQ, Conners Teacher's Abbreviated Symptom Questionnaire; NR, not reported*.

A prospective study assessed effects of acute methylphenidate discontinuation on neuropsychological performance, as evaluated by the Cambridge Neuropsychological Test Automated Battery (CANTAB) test battery, in 15 youth ages 4 to 14 with ADHD (27). Prior to assessment, youth were stabilized on MPH for at least 3 months. For initial testing, youth discontinued MPH for at least 24 h, and retest occurred after resuming for more than 1 week. Three tests (spatial recognition, spatial span, and delayed matching to sample) showed no significant difference between testing conditions, and three tests (pattern recognition, spatial working memory, and intra/extra dimensional set-shifting) showed superior performance while taking MPH. The differences in subtest results were suggestive of MPH treatment effects on executive function in ADHD. All except one youth was clearly rated as more symptomatic for ADHD symptoms while off MPH. Limitations include small sample size and that youth were tested after having been off methylphenidate for only a short time period.

The other observational study completed a prospective, non-controlled trial on 42 youth, followed more than 1 year (13 followed 2 years) after diagnosis of ADHD and treatment with MPH (28). Each youth was given up to three different doses of MPH and placebo × 1 month, with the primary outcome measure being their schoolteacher-rated Conner's Abbreviated Symptoms Questionnaire (ASQ). Of 28 youth who had completed a randomized placebo month, they found that 17 relapsed including 5 whose functioning deteriorated so significantly they could not sustain the protocol's full month of placebo treatment. Eleven remained with Conner's ASQ score <15 on switch to placebo, with undetectable change in functioning to their schoolteachers. Sustained remission off medication was not predicted by age or IQ. Only 2/3 of sample had completed the trial off medication by time of publication and only teacher-rated outcomes were measured. Authors recommended periodic drug-free trials to assess for ongoing need for medication.

In summary, short-term (>24 h) discontinuation of MPH affected youth performance on neuropsychological testing. Worsening of teacher-rated ADHD symptoms was seen in a majority (60%) but not all youth who were given placebo for 1 month during treatment with methylphenidate.

### Case Reports

A number of case reports describe events related to discontinuation of stimulant medications and provide guidance for the clinician (Table 3). Four separate publications describing seven children report acute dystonic reactions after stopping psychostimulants in children on concurrent antipsychotic medications. The stimulants involved include both methylphenidate and amphetamine products, co-prescribed with antipsychotic medications, risperidone, and aripiprazole. In five of the cases, the dystonia onsets within 33 h and within 10 days in two cases. The proposed mechanism is stimulants enhanced synaptic levels of dopamine and their sudden cessation removed a counter to dopamine blockade by antipsychotic and allowed enhanced binding to striatum. Treatment with anticholinergic agents or restarting the stimulants resolved the dystonic movements. A gradual taper of stimulants with careful vigilance for abnormal movements is suggested if stopping stimulants in this context (29–32).

**Table 3:** Case reports of discontinuation of psychostimulants in children with ADHD.

**Study**	**Age and sex**	**Medication**	**Discontinuation**	**Adverse reaction**	**Clinical pearls**
Benjamin 2005	9-year-old M 9-year-old M 13-year-old M	MPH 15 mg TID, risperidone 1.5 mg TID, clonidine 0.1 mg QHS, valproic acid 250 TID Dextroamphetamine racemic 10 mg TID, risperidone 1 mg BID, clonidine 0.1 mg QHS, valproic acid 125 mg qam and 250 mg QHS Fluvoxamine 150 mg BID, MPH 54 mg/day, guanfacine 1 mg BID, risperidone 0.5 mg TID	MPH stopped suddenly AMP stopped suddenly Missed MPH dose over 1 day	Observed dystonic reaction resolved with benztropine Observed dystonic reaction resolved with benztropine Dystonic reaction resolved on own in 24 h after restarting MPH	Sudden discontinuation of stimulant medication used concomitantly with an antipsychotic may lead to acute dystonic reactions.
Guler 2015	9-year-old M	MPH 54 mg, risperidone 1.5 mg BID	Missed dose of stimulant	Dystonic reaction observed 6–7 h following missed dose	
McLaren 2010	11-year-old M	Aripiprazole 15 mg BID, OROS MPH 108 mg qam, lithium 600 mg qam and 300 mg QHS, clonidine 0.2 mg BID	Abrupt cessation of OROS MPH	Acute dystonic reaction 33 h after last dose that resolved with IM diphenhydramine	
Parraga 2015	9-year-old F 7-year-old M	MPH CD 50 mg qam and MPH 5 mg every afternoon, aripiprazole 1 mg BID Dextroamphetamine-racemic 30 mg/day and aripiprazole 2 mg daily	Abrupt cessation of MPH CD and MPH Abrupt cessation of dextroamphetamine	Dystonic reaction occurred that responded to diphenhydramine and discontinuation of aripiprazole Dystonic reaction occurred several days after and resolved with decrease in SGA and restarting stimulant	
Connor 1995	9-year-old M	Perphenazine 16 mg/day, dextroamphetamine 40 mg/day, fluoxetine 20 mg/day, diphenhydramine 50 mg/day	Perphenazine tapered by 4 mg/day, then stopped. Fluoxetine and diphenhydramine suddenly stopped without tapper. Dextroamphetamine continued.	AIMS score became elevated at day 2 from discontinuation of the perphenazine and continued to worsen after 10 days off of the antipsychotic. Stimulant was tapered over 2 days with rapid improvement in AIMS score	Concomitant use of stimulant may increase risk for neuroleptic withdrawal dyskinesias on stopping antipsychotics
Connor 1998	11-year-old M	MPH 10 mg BID, thioridazine 150 mg/day,	Thioridazine tapered over 3 weeks, MPH continued	One week after stopping thioridazine, increase in abnormal muscle movements and AIMS elevation.	
Hollis 2007	7-year-old M	Risperidone 1.5 mg, MPH 36 mg	Abrupt discontinuation of risperidone and subsequent initiation of MPH 36 mg 12 h later	Within 8 h, dyskinesias observed that resolved with restarting risperidone	
Bernard 2015	16-year-old M	Long-term MPH at 30 mg/day	MPH stopped suddenly	After stopping MPH, dramatic increase in weight gain and subsequent development of an eating disorder	Cessation from stimulant medications may cause withdrawal symptoms that impacting GI and neuromuscular systems.
Cuskun 2013	13-year-old F	IR MPH 20 mg qam	Missed MPH dose	Painful muscle cramps 24 h after missed dose of IR MPH. Switched to OROS MPH and cramps resolved on drug-free days	
Krakowski 2018	11-year-old F	1st Trial—OROS MPH 36 mg 2nd Trial—Lisdexamfetamine 50 mg/day, guanfacine ER 3 mg/day, fluoxetine 20 mg/day.	1st—Abrupt cessation of OROS MPH 2nd—Taper off lisdexamfetamine by 10 mg	1st—Acute vomiting and light sensitivity noted following cessation of OROS MPH. 2nd—Reduction in stimulant caused migraines and malaise for a 2-day period following each reduction	

Withdrawal dyskinesias with discontinuation of antipsychotic in children on stimulants are reported in three other case reports. The mechanisms in this case are also felt to reflect competing actions on the dopamine system by antipsychotic medications and stimulants. The abnormal movements were precipitated by sudden or gradual withdrawal of the antipsychotic. In these cases, it appears that the dyskinesias can persist for several weeks after the stimulants are stopped. To minimize this adverse effect, the authors suggested a gradual taper over several weeks of the antipsychotic with prompt discontinuation of the stimulant if abnormal involuntary movements appear (33–35).

Additional case reports highlight withdrawal symptoms that impact both the gastrointestinal and neuromuscular systems. One report involves a 13-year-old female who developed painful muscle cramps in her legs when immediate-release methylphenidate was stopped for a summer holiday. Cramps occurred in the morning after a drug-free day (36). Acute withdrawal symptoms indicating tolerance are reported in a 11-year-old female with ASD who developed vomiting, headaches, light sensitivity, and malaise following abrupt discontinuation of MPH and dose reduction of lisdexamfetamine. Children with ASD may be more sensitive to stimulant medications (37). A case of a child who gained a significant amount of weight and eventually developed an eating disorder after stopping stimulants is reported (38). These case reports provide guidance to the clinician for managing potential adverse effects related to stopping medications in a child treated with both products.

Several case reports describe mental health adverse effects from stopping psychostimulants. Psychiatric complications are reported in two case reports; the first involved severe depression in a child taken off pemoline (39), and the second reports on a psychotic manic-like appearance within 7 days in a child taken off methylphenidate (40). Finally, three case reports describe how behavioral interventions were used to address behaviors and allow discontinuation of stimulants in children with ADHD. In these cases, intensive behavioral management therapies to parents and teachers were able to successfully manage behaviors as stimulants were gradually tapered and stopped (41–43).

These case reports provide guidance to the clinician for managing potential adverse effects related to stopping medications in a child treated with both products.

### Clinical Guidelines and Literature Reviews

While confirming the efficacy of psychostimulants for ADHD, three national guidelines for the evaluation and treatment of ADHD suggest consideration of periodic trials of stopping medications to determine continued need. Discontinuing ADHD medications in children requires a plan to monitor for return of symptoms (44). The AACAP Practice Parameter on ADHD suggests in general continuing medication through adolescence due to a high level of maladaptive behavior in patients with ADHD. It further clarifies that if a patient has been symptom free for 1 year, then it is appropriate to consider stopping the medication. Factors that may support discontinuation of medications include no recent need for dose adjustment and lack of deterioration with missed doses or drug holidays (5). Similarly, the National Institute for Health and Care Excellence guidelines for ADHD suggests an annual review of whether medications should be continued. The assessment includes the preference of the youth and family, current benefits of the medications, adverse effects, clinical need, impact on education and employment, effects of missed doses, and need for additional supports (45).

A systematic literature review of 53 articles on how long to treat ADHD provides additional support for the efficacy of treating youth with ADHD with medications for up to a period of 2 years. There is limited evidence for the long-term advantage of medications beyond “mere” symptom control, and information on long-term adverse effects is limited. While acknowledging the substantial clinical experience that many children with ADHD continue to benefit from long-term medication treatment to control symptoms, the authors support annual medication free periods lasting several days to 1 week to determine ongoing benefit (46).

An additional literature review addresses the use of drug holidays as a procedure to minimize or reverse adverse effects of these medications, (e.g., growth retardation, weight loss). This report found 22 studies surveying drug holidays to manage side effects such as child growth and insomnia or reduce tolerance of medications. The review finds the practice to be common in 25 to 70% of families. The practice of drug holidays can be useful as a periodic trial of medication discontinuation to manage risk: benefit ratio and increase voice of youth and families to guide treatment. However, provider's opinions on the value of drug holidays are mixed and more evidence is needed (47).

## Discussion

This review has implications in several areas for consumers and clinicians addressing optimal duration of stimulant treatment for youth with ADHD and potential outcomes if medications are stopped. Few trials set out to answer the question of “how/when/should we discontinue stimulants for ADHD?,” so this review attempted to synthesize the available information to help answer this question. Nevertheless, there are important points to guide the consideration of discontinuation.

All reviewed randomized withdrawal studies support the use of medications to reduce symptoms, improve quality of life, or reduce relapse rates. Most studies show early re-emergence of ADHD symptoms for most children discontinuing stimulants. Despite these considerations, there is a significant subpopulation of youth in these RCTs (~30%) who may tolerate discontinuation without relapse of ADHD (17, 22, 25). A similar observational study that studied 1-month placebo trials in children with ADHD found that 11 out of 42 children (26%) showed no clinical deterioration when medication was stopped (28). One RCT suggested that older youth were less likely to have symptom recurrence than younger youth, with those older than a median age of 13.8 years showing no worsening when switched to placebo (22).

One limitation of these RCTs is that several are industry sponsored, designed to evaluate the efficacy of medications and may be biased toward children who have shown significant early responses and good tolerance to medication (14, 16, 17, 26). Often, the population randomized for possible discontinuation have had lengthy lead-in periods of successful treatment with stimulants. A typical clinical population may experience less robust response or suffer more side effects, altering cost–benefit considerations. With one exception (19), the placebo phase of the discontinuation trials is brief so rates of relapse may not compare equally with those seen in a community population. Trials are either exclusively boys or majority boys and the clinical significance of some RCT differences between active drug and placebo can be questioned.

In addition to controlled intentional discontinuation studies, analyses of administrative databases can offer insights into continued ADHD medication effects. These studies compare outcomes during periods after prescriptions are filled to periods when prescriptions are not filled. After filling ADHD medication prescriptions, youth are less likely to suffer from unintentional injuries and substance-related events and visit EDs for unintentional injuries including traumatic brain injuries and trauma-related events (48–52). In Sweden, after filling ADHD prescriptions, young people scored higher on college entrance exams and adolescents and adults underwent fewer criminal convictions, while filling SSRI prescriptions showed no effects (53, 54). Limits of database studies to consider are the fact that they are associational, cannot imply causation, and carry a risk of selection bias. However, these studies support the continued effect of treatment for ADHD to reduce injuries, motor vehicle crashes, criminality, and substance abuse. ADHD medication prescriptions are not filled for many reasons, so these studies were not included in our search, but may help inform stakeholder cost–benefit considerations when contemplating medication discontinuation.

The long-term observational MTA study may offer clues to identifying children with ADHD who may tolerate discontinuation of stimulants. Latent classes were identified by the trajectories of long-term response to treatment, and by 6 to 8 years, the type of treatment at 14 months did not predict functioning (55). Adherence was an issue during follow-up; 62% of cohort had stopped medication or not on medication at 8-year follow-up. By then, three trajectories of ADHD were proposed, illustrating a natural course of disease. “Class 1” showed a gradual improvement with increasing benefit of medication at 3 years, “class 2” showed a larger initial improvement with medication maintained over time, and “class 3” showed an initial positive response to medications and then a return to pretreatment levels (56). It is possible that a careful consideration of risks and benefits of continued stimulant treatment in youth matching a “class 3” subtype could lead to a decision to discontinue stimulants.

Brain maturation and/or the development of compensatory strategies may facilitate discontinuation of medications for ADHD. As children age into adults, working memory, planning, and problem solving become more efficient (57). Similarly, as children with ADHD age, measures of executive function improve (58). The age and overall maturity of the individual are additional variables in a decision to discontinue stimulants. One may expect psychosocial functioning of children with ADHD to improve with therapy. Three RCTs examining the effect of behavioral therapies did not show benefit of behavioral therapies to augment discontinuation of stimulants but case reports suggest such an approach may succeed.

Deprescribing as a systematic approach to providing the minimum effective dose or number of medications identifying can be applied to youth with ADHD. The strength of the diagnosis and natural course of the condition along with previous responses to psychostimulants are considered along with an assessment of their risks and benefits. Periodic discontinuation trials of psychostimulants in ADHD are supported by the practice guidelines (5, 45, 59). While an annual medication free trial is suggested, stopping stimulants is a clinical decision made on an individual basis considering many factors (46).

It is important to have a shared decision discussion on whether stimulants should be continued with the family and youth in which one reviews comorbid conditions, adverse effects, timing with school or other important events, and if they still feel the medications are needed (19, 22). Differences in medication discontinuation in minority youth have been reported, so it is important to evaluate the discussion within the context of racial and ethnic disparities (60). The setting and expectations of the child during a discontinuation period are also important considerations as measures of increased ADHD symptoms may differ between home and school (25).

The drug holiday trials support intentional short periods of discontinuation to not only identify youth who may no longer require medications to succeed but manage side effects as well. Stimulants tend to have quick onset of action and short half-life allowing for short-term “drug holidays,” such as weekends or school holidays, to mitigate adverse effects including growth retardation, weight loss, and insomnia (20, 24, 47). Weekend drug holidays were not shown to effect school performance (21) and planned drug holidays also help clinicians identify candidates for discontinuation (61). Drug holidays are well accepted by parents (24). Periodic trials of stimulant discontinuation are also supported by long-term observational studies that show that consistent stimulant treatment of 16 years is associated with significant decreases in height and increases in weight (62). This suggests that some youth will enjoy relief from growth deficits with intermittent use.

The literature supports careful monitoring as necessary for any discontinuation trial. Many of the case reports describe the emergence of side effects associated with stopping stimulants used to treat ADHD and provide clinical guidance on safety of discontinuation or withdrawal of medications. Temporary movement disorders may emerge when stimulants are discontinued and may occur more frequently with concomitant antipsychotics. Psychostimulants have been associated with motor tics, but in RCT, abrupt withdrawal of medications did not exacerbate tics (23) and led to no rebound or withdrawal symptoms (16). Clinicians also need to consider whether the stimulants are short-acting or long-acting as adverse effects associated with discontinuation may differ between drug types with different half-lives (63).

In most cases, relapse is noticed within 2 weeks, so a discontinuation trial could be brief (17, 18). One RCT lays out a potential plan for discontinuation suggesting a 1-week drug-free trial with assessment of the child in multiple different settings. If this is inconclusive, the authors suggest a placebo-controlled trial of stimulants lasting 3–4 weeks and consideration of higher dose (25). However, a conservative approach would suggest a gradual taper of a medication used to treat ADHD over the course of several weeks to months to reduce the likelihood of immediate adverse effects and rapid symptom reemergence.

## Limitations

While the recent inclusion of deprescribing as a PubMed search term provided some reference, a comprehensive search of the desired search was difficult to design, and studies with relevant data often used different descriptors. By necessity, the review evolved from the systematic review to a targeted review as initial results revealed little guidance on informing the question of deprescribing or planned discontinuation of stimulants. It is possible that some relevant articles have not been located. Also, our focus was on intentional discontinuation of stimulants by providers so literature on patient adherence with ADHD medications was not included in the review (13). Due to this restriction, our identified studies may include more cooperative families and less impaired subjects and alter the focus of this paper from typical treatment experiences.

Industry sponsorship and study design may influence the findings. Six RCTs were sponsored grants. Three of the RCTs were sponsored by pharmaceutical companies, and these coincided with the three of the four largest RCTs (*n* ≥ 75) and showed discontinuation of stimulants (with or without therapies), resulting in rapid re-emergence of ADHD symptoms. Also, the study populations included many more males in their cohorts.

## Conclusions

This systematic review summarizes the literature on deprescribing stimulants for ADHD in children and adolescents, in particular characterizing rates of symptom re-emergence and medication withdrawal-emergent side effects. In summary, our review indicates that a significant group of youth may tolerate discontinuation of stimulants, but more research is needed to clearly understand and identify them. Further research is also needed to determine the optimal duration of treatment and inform evidence-based guidelines for discontinuation when appropriate.

## Author Contributions

WL, JW, MB, EG, WM, DC, and TL were involved in the conception and design of the work, which includes drafting and revising the intellectual content. All authors approve the version now submitted and agree to be accountable for all aspects of the work.

## Conflict of Interest

The authors declare that the research was conducted in the absence of any commercial or financial relationships that could be construed as a potential conflict of interest.
